# Supplementary material for: Factors Contributing to Breastfeeding Cessation Among Arab Women in Israel
Source: Nutrients. 2025 Feb 19;17(4):735. doi: 10.3390/nu17040735 (PMC11858111; doi:10.3390/nu17040735)
Supplement: Supplementary file 1 [file nutrients-17-00735-s001.zip › nutrients-3391058-supplementary.pdf]

Supplementary S1. Informed Consent Form for Online Studies

Part I: Informed Consent Form for Online Studies

Greetings,

You are invited to participate in a study entitled "Characteristics and Factors Israeli Arab Mothers Related to Exclusive Breastfeeding. This study was conducted by researchers from the Ramat Gan Academic College and The Academic College of Tel Aviv-Jaffa. We thank you for dedicating your time and participating in the study.

The purpose of the study is to investigate the factors associated with exclusive breastfeeding amongst Arab mothers living in Israel.

The purpose of the study to investigate the factors predicting Exclusive breastfeeding among Arab mothers living in Israel

The time required to fill out the questionnaire is about 15 min. The questionnaire is anonymous and will be filled out anonymously, your answers are completely confidential, and will not be used in any way except for research purposes.

In this questionnaire, you will be presented with several different questions regarding the effect of treatments on the quality of life and emotional reactions of transplant recipients. You are asked to mark the correct answer next to each question.

You do not have to answer all the questions. If you feel uncomfortable, you may stop filling out the questionnaire at any stage.

By agreeing to fill out this questionnaire, you declare that you are over 18 years old.

It is assumed that participating in the research will not bring you any personal profit or advantage, but we hope that your participation will contribute to general knowledge in this research field.

It should be noted that we are not aware of any risks by participating in the study, but as with any online activity there is a certain risk of breach of privacy. We make every effort to reduce this risk by have the questionnaire filled out anonymously and not using the details except for the purpose of the study.

At the end of the study, you can get more information from the Ethics Committee of the Tel Aviv-Jaffa Academic College using the Debriefing form. In addition, you can contact the research team by email:

We thank you very much for filling out the questionnaires in full.

Regards,

Dr. Khaled Awawdi : Awawdi.h@iac.ac.il Dr. Tarabia Mahdi: mahdita@mta.ac.il

By clicking the "I agree" button, you express your consent to participate in the study. By clicking on the "I do not agree" button, you terminate your participation in the study.

Supplementary S2

Part A: Socio-Demographic and Employment Characteristics

1. Age? ___________________
2. Marital Status:
    1. Married or living with a partner
    2. Single
    3. Divorced or separated
    4. Widowed
3. What is your nationality?
    1. Arab Muslim
    2. Arab Christian
    3. Arab Druze
4. Region?

    1. North
    2. Central
    3. South
5. Level of Religiosity. Please circle the extent to which you agree with the following statements. 1 = Strongly disagree, 5 = Strongly agree.
    1. Do you believe in religious values?
    2. Do you behave according to traditional religious values?
    3. Do you observe the commandments of your faith?
6. How many children do you have from before your last birth? (Not including your most recent child)
    1. 0
    2. 1
    3. 2
    4. 3
    5. 4 or more
7. How many people live in your household permanently?
    1. 1
    2. 2
    3. 3
    4. 4 or more
8. What is your level of education?
    1. Primary
    2. Secondary
    3. Academic
9. Did you work before the birth or until shortly before it, and what are the characteristics of your job?
    1. I did not work
    2. Self-employed
    3. Employed
10. Did you work before the birth or until shortly before it, and what was the extent of your work? (If you are employed at a different percentage than listed below, please mark the higher percentage.)
    1. I did not work
    2. I worked, full-time 100%
    3. I worked, 75% part-time
    4. I worked, 50% part-time
    5. I worked, 25% part-time
11. Do you intend to return to work?
    1. Yes
    2. No
    3. I don't know
    4. I have already returned to work
12. At what age of the baby do you intend to return to work or have you returned to work?
    1. 1 month
    2. 2 months
    3. 3 months
    4. Between 4 to 6 months
    5. 6 months
    6. I do not plan to return to work

13. The average income for a family recently measured is between ILR12,000 and ILR15,000. How would you rate your family's income?
    1. Below average
    2. Average income
    3. Above average

Part B: Details on Infant Feeding

Exclusive breastfeeding is defined as the natural feeding of breast milk or expressed breast milk without any supplementation of infant formula (IF), including any formula given in the hospital after birth.

1. During your prenatal follow-up or your stay in the hospital, did you receive guidance on breastfeeding, and in what form?
    1. I did not receive any conversation or guidance on breastfeeding.
    2. I received a group conversation or guidance on breastfeeding.
    3. I received individual conversation or assistance regarding breastfeeding.
2. What was the nature of the feeding your infant received since the last birth in the past four months?
    1. Infant formula (IF) only.
    2. Partial/combined breastfeeding combined with IF.
    3. Exclusive breastfeeding (breast milk and/or expressed breast milk) only.
3. If your infant's feeding method in the past four months was based on exclusive breastfeeding, how long did the breastfeeding last?
    1. I did not breastfeed at all.
    2. Up to one month.
    3. Up to two months.
    4. Up to three months.
    5. Up to four months.
    6. More than four months.
4. If the feeding method for your baby over the past four months, or part of that time, was based on exclusive breastfeeding, please indicate the degree of influence each of the following factors had on your decision to breastfeed:

Factors Affecting Decision to Breastfeed		Did Not Impact My Decision	Had Some Impact on My Decision	Had Significant Impact on My Decision
1.	Family Members (Mother, Mother-in-law, Sisters)	0	1	2
2.	Health Clinic Nurse	0	1	2
3.	Hospital Staff	0	1	2
4.	Childbirth Preparation Course	0	1	2
5.	Successful Previous Experience with Breastfeeding a Previous Child	0	1	2
6.	Economic Consideration (Breast Milk is Free)	0	1	2
7.	Desire for a Good Bond with the Child	0	1	2
8.	Maintaining the Health of Your Baby	0	1	2
9.	Information from the Internet, Literature, and Media	0	1	2

5. If the feeding method for your baby over the past four months, or part of that time, was based on exclusive or partial breastfeeding, please indicate for each of the following reasons whether it was a reason for you to stop breastfeeding:

Reasons Affecting Decision to Stop Breastfeed		Was NOT a Reason to Stop	WAS a Reason to Stop
1.	Because you did not have enough milk or the milk was not good enough, and the baby constantly needed formula supplementation.	0	1

2.	Because of health issues of the baby: illness, hospitalization, or medication that prevented them from breastfeeding.	0	1
3.	Because of your own health issues: illness, hospitalization, or medication that prevented you from breastfeeding.	0	1
4.	Due to fatigue, discomfort, lack of time, or dissatisfaction with the breastfeeding experience.	0	1
5.	Due to lack of support and guidance from professionals for continuing breastfeeding.	0	1
6.	Because returning to work and work conditions were not supportive enough for continuing breastfeeding or expressing milk.	0	1
